# The TIP60-CD44 Axis Modulates Colorectal Cancer Stemness

**DOI:** 10.3390/cells14100686

**Published:** 2025-05-09

**Authors:** Asad Mohammad, Sudhakar Jha

**Affiliations:** Department of Physiological Sciences, College of Veterinary Medicine, Oklahoma State University, Stillwater, OK 74078, USA; moasad@okstate.edu

**Keywords:** colorectal cancer, cancer stem cells, epithelial–mesenchymal transition, CD44

## Abstract

HIV-1 Tat-interactive protein of 60 kDa (TIP60) is a lysine acetyltransferase protein that can acetylate histone and non-histone proteins. This study highlights TIP60’s role in regulating colorectal cancer (CRC) stemness. The depletion of *TIP60* resulted in a marked decrease in cellular proliferation, highlighting TIP60’s involvement in the progression of CRC. Additionally, the loss of TIP60 impacted colony formation, transitioning from densely packed structures to dispersed spindle networks along with the loss of E-cadherin, indicating its role in the epithelial–mesenchymal transition (EMT). Three-dimensional culture models suggest that TIP60 is vital for spheroid formation, highlighting its importance in maintaining cancer stem cell properties in CRC. *TIP60*-depleted cells showed increased invasion in a 3D basement membrane extract (BME) invasion matrix, demonstrating its essential role in cellular invasiveness. Mechanistically, the reduction of TIP60 resulted in a decrease in *CD44* expression, a critical marker for cancer stem cells (CSCs). Notably, *CD44* overexpression restored the efficiency of spheroid formation and cell proliferation while reversing the EMT phenotype. Developing the TIP60-CD44 axis as a therapeutic target to treat CRC stemness and metastasis will help decrease the burden due to the deadly disease.

## 1. Introduction

Colorectal cancer (CRC) is the second leading cause of cancer-related mortality worldwide [1]. Advancements in therapeutic strategies have significantly improved the survival rates of patients diagnosed with CRC [2]. Despite the development of innovative targeted medications and several therapeutic combinations, tumor recurrence and drug resistance remain significant obstacles to conventional, targeted, and immunotherapeutic therapies [3]. Colorectal cancer stem cells (CRCSCs) are the primary determinant of cancer development, recurrence, and dissemination [4]. Cancer stem cells (CSCs) are a distinct group of cells in the tumor bulk that can renew themselves and differentiate into other types of cells [5]. CSCs have a role in tumor recurrence, metastasis, heterogeneity, and multidrug resistance [6,7].

HIV-1 Tat-interactive protein (TIP60), also known as lysine acetyltransferase 5 (KAT5), was first discovered as a cellular acetyltransferase protein interacting with HIV-1 Tat [8]. TIP60 belongs to the MYST (Moz, Ybf2/Sas3, Sas2, and TIP60) family of acetyltransferases and has been shown to acetylate histones [9] and non-histone proteins [10,11]. The MYST family members regulate chromatin remodeling, gene-specific transcription, and DNA damage repair [12]. *TIP60* downregulation has been associated with several cancer types, such as colorectal, gastric, and breast cancer [13]. *TIP60* has been shown to regulate cellular migration and invasion [14] and inhibit epithelial–mesenchymal transition (EMT) [15]. Apart from cancer, *TIP60* has also been shown to be involved in regulating stem cell functions. In hematopoietic stem cells (HSCs), *TIP60* is essential for its maintenance [16]. The *TIP60* histone acetyltransferase in embryonic stem cells (ESCs) triggers the activation of genes necessary for cell division and suppresses genes that induce differentiation [17]. CD44 belongs to the cell adhesion molecule (CAM) class and is a single-chain transmembrane glycoprotein. *CD44* is implicated in various types of cancers, such as breast cancer, prostate cancer, colorectal cancer, and pancreatic cancer. *CD44* is involved in tumor growth, EMT, cellular invasion, metastasis, and resistance to chemotherapy [18]. It is an established marker for CSCs [19].

There remains a lack of knowledge regarding the role that *TIP60* plays in the control of cancer stemness. Our research sheds light on the significance of *TIP60* in the stemness of CRC, demonstrating that *TIP60* affects the cancer stemness characteristics in CRC. Mechanistically, we identified that *TIP60* controls cancer stemness via CD44.

## 2. Materials and Methods

### 2.1. Cell Culture

Colorectal cancer cell lines HCT116 (Cat. No. CCL-247) and 293T (Cat. No. CRL-3216) were purchased from ATCC (Manassas, VA, USA)and were grown in DMEM High glucose (Gibco, Thermo Fisher Scientific, Waltham, MA, USA; Cat. No. 11-995-081) supplemented with 10% Fetal Bovine Serum (Corning, NY, USA; Cat. No. 35-010-CV).

### 2.2. Generation of Stable Cell Line

An shRNA designed against the *TIP60* (TOP 5′-CTGATCGAGTTCAGCTATGAACTCGAGTTCATAGCTGAACTCGATCAG-3′, Bottom 5′-CAAAAACTGATCGAGTTCAGCTATGAACTCGAGTTCATAGCTGAACTCGATCAG-3′) was cloned into a pLKO vector using AgeI and EcoRI restriction sites. For the amplification of the plasmids, the construct (*TIP60*-targeting, Sh*TIP60*, and control vector, ShLuc) plasmids were transformed into Stbl2 cells. The lentivirus was generated by transfecting 5 × 10^6^ 293T cells with *TIP60*-targeting (Sh*TIP60*) and control vector (ShLuc) plasmids using polyethylenimine (Fisher Scientific, Waltham, MA, USA; Cat. No. 24314-2) with a 1:3 ratio and incubated for 24 h. After 24 h, the media were changed, and fresh medium was added and incubated for 48 h. After 48 h, the virus was harvested and used to infect 2 × 10^6^ HCT116 cells with polybrene (Millipore Sigma, Burlington, MA, USA; Cat. No. TR1003) reagent (1 mg/mL). After 24 h, the media containing the virus were replaced with fresh growth media, and puromycin (Fisher Scientific, Waltham, MA, USA; Cat. No. AAJ672368EQ) (5 μg/mL) was added to the growth media for selection. The cells were selected until the mock-transfected cells died.

*TIP60* wild-type (*TIP60*_WT) and *TIP60* catalytic dead (*TIP60*_KD) were generated by retrovirus transfection to 5 × 10^6^ 293T cells with the plasmids (*TIP60* wild-type (*TIP60*_WT) and *TIP60* catalytic dead (*TIP60*_KD)) utilizing Lipofectamine 2000 (Invitrogen, Thermo Fisher Scientific, Waltham, MA, USA; Cat. No. 11668019) following the manufacturer’s instructions. The virus was collected 72 h post-transfection and used to infect 2 × 10^6^ HCT116 cells with polybrene at a 0.4 mg/mL concentration. After 6 h, the virus-containing media were substituted with growth media. Puromycin (5 μg/mL) was added to the growth medium for selection after 24 h. The cells were selected until the mock-transfected cells died, and the puromycin-selected cells were maintained for two weeks to generate stocks.

### 2.3. CD44 Overexpression

To overexpress *CD44* in HCT116 cell lines, a plasmid containing the full-length *CD44s* (Cat. No.137812) gene was obtained from Addgene (Watertown, MA, USA). HCT116 control (ShLuc) and *TIP60*-depleted (Sh*TIP60*) cells were placed in 6-well plates. When the cell culture reached 60% confluency, the plasmid (1:3) was introduced into the cells using polyethylenimine. After 72 h post-transfection, the media were substituted with a fresh medium. The cells were selected utilizing neomycin (1 mg/mL) (Research Product International, Mount Prospect, IL, USA; Cat. No. N20040-25).

### 2.4. RNA Isolation and Quantitative PCR (qPCR)

HCT116 control (ShLuc) and *TIP60*-depleted (Sh*TIP60*) cells were on a 10 cm dish. After reaching 70% confluency, the cells were trypsinized (Corning, NY, USA; Cat. No. 25-053 CI) and counted utilizing a hemacytometer (Fisher Scientific, Waltham, MA, USA; Cat. No. 22-600-100). One hundred thousand HCT116 control cells (ShLuc) and *TIP60*-depleted (Sh*TIP60*) cells were seeded into 6-well ultra-low attachment plates (Corning, NY, USA; Cat. No. 07-200-601) and incubated for 7 days. The cells were collected and washed with ice-cold PBS, and the whole RNA was extracted using an RNeasy Kit (Qiagen, Hilden, Germany; Cat. No. 74104), following the manufacturer’s instructions, and reverse-transcribed to cDNA using the iSCRIPT cDNA synthesis kit (Bio-Rad Laboratories, Hercules, CA, USA; Cat. No. 1708891). Quantitative real-time PCR (RT-qPCR) was performed using iTaq Universal SYBR Green Supermix (Bio-Rad Laboratories, Hercules, CA, USA; Cat. No. 1725124) on Quant Studio 6 Pro (Applied Biosystem, Thermo Fisher Scientific, Waltham, MA, USA). The amplification conditions were as follows: 50 °C for 2 min, 95 °C for 5 min; 40 cycles of amplification (95 °C for 15 s, 60 °C for 1 min); and the melt curve step (95 °C for 15 s, 60 °C for 1 min, 60 °C → 95 °C (0.1 °C/s)). The mRNA expression of the gene was normalized to the average of the two housekeeping genes *ACTB* and *GAPDH*. Gene expression analysis was performed using qPCR and computed using ΔΔCt. The ΔCt was calculated by subtracting the housekeeping gene’s Ct value from the target gene’s Ct value (ΔCt = Ct_target − Avg Ct_housekeeping, *ACTB* and *GAPDH*). To compute ΔΔCt, the values were subtracted from the control sample’s ΔCt from each experimental sample’s ΔCt (ΔΔCt = ΔCt_experimental − ΔCt_control). Finally, gene expression in fold change was calculated using the method 2^−ΔΔCt^ [20]. The primers utilized for detecting mRNA expression levels were purchased from Integrated DNA Technologies (IDT), and the sequences of each forward primer (F) and reverse primer (R) are as Table 1.

### 2.5. Immunofluorescence

Cells were grown in 6-well plates (Corning Costar, NY, USA; Cat. No. 07-200-83) on 15 mm coverslips. At 70% confluency, the cells were fixed in 100% ice-cold methanol for 15 min. Cells were blocked in 3% BSA (Sigma-Aldrich, St. Louis, MO, USA; Cat. No. A2153) for 1 h and incubated with mouse anti-E-cadherin (BD Biosciences, San Jose, CA, USA; Cat. No. 610182) primary antibody at a 1:200 ratio for 1 h at room temperature. Cells were washed twice with PBS, and the secondary antibody, goat anti-mouse (Invitrogen, Thermo Fisher Scientific, Waltham, MA, USA; Cat. No. A11029), was added at a 1:1000 ratio and incubated for 1 h. After incubation, the cells were washed twice with 1× PBS. The stained cells were then mounted onto the glass slides using DAPI (Vector Laboratories, Newark, CA, USA; Cat. No. NC1848443). Images were captured using LSM 980 with Airyscan 2 confocal laser scanning microscope (Carl Zeiss Microscopy GmbH, Jena, Germany).

### 2.6. Cell Invasion Assay

Two hundred HCT116 Sh*TIP60* and ShLuc cells were seeded into a 96-well ultra-low attachment round-bottom plate (Corning, Cat. No. 07-201-680). Immediately after seeding, the cells were centrifuged at 1200 rpm for 5 min and incubated for 72 h for spheroid formation. After 72 h, 100 µL of BME invasion matrix (R&D Systems, Minneapolis, MN, USA; Cat. No. 3500096K) was added to each well. The cells were further incubated for 72-96 h. Images were taken at 10× magnification on a BioTek Cytation 5 imager (Agilent Technologies, Santa Clara, CA, USA).

### 2.7. Spheroid Formation Assay (3D Culture)

HCT116 control (ShLuc) and *TIP60*-depleted (Sh*TIP60*) cells were cultured in a 10 cm dish. After reaching 70% confluency, the cells were trypsinized (Corning, Cat. No. 25-053 CI) and counted utilizing a hemacytometer (Fisher Scientific, Waltham, MA, USA; Cat. No. 22-600-100). One hundred cells were seeded in each well of 96-well flat ultra-low attachment plates (Corning, NY, USA; Cat. No. 07-200-603). The cells were incubated for 7 days, and images were taken using a BioTek Cytation 5 imager (Agilent Technologies, Santa Clara, CA, USA). For live and dead cell staining of spheroid culture, Calcein AM (Thermo Fisher Scientific, Waltham, MA, USA; Cat. No. C1430) and Ethidium Homodimer-1 (EthD1) (Thermo Fisher Scientific, Waltham, MA, USA; Cat. No. E1169) were added at a concentration of 1 μg/mL. The cells were incubated for 30 min at 37 °C in a cell culture incubator. After the incubation, images were taken using a BioTek Cytation 5 imager (Agilent Technologies, Santa Clara, CA, USA).

### 2.8. Colony Formation Assay and Absorbance Assay

HCT116 control (ShLuc) and *TIP60*-depleted (Sh*TIP60*) cells were grown in a 10 cm dish. The cells were trypsinized and counted using a hemacytometer after 70% confluency. One thousand HCT116 control (ShLuc) and *TIP60*-depleted (Sh*TIP60*) cells were seeded in 6-well plates and incubated for 11 days. On day 11, cells were fixed with a 0.5% Crystal Violet (Fisher Scientific, Waltham, MA, USA; Cat. No. NC0827019) staining solution. The cells were washed twice with 1× PBS, and 250 µL of Crystal Violet solution was added to each well for 5 min. After incubation, the cells were washed three times with PBS. The plates were air-dried, and images were taken using a BioTek Cytation 5 imager (Agilent Technologies, Santa Clara, CA, USA). For the absorbance assay, 1% SDS (Bio-Rad Laboratories, Hercules, CA, USA; Cat. No. 1610302) was used to destain Crystal Violet. Five hundred microliters of 1% SDS were added to each well of 6-well plates and incubated for 6 h on a shaker at room temperature. After the incubation, 100 μL of the destained solution was added to 96-well plates for HCT116 ShLuc and Sh*TIP60* cells. Absorbance readings were taken at 590 nm on a BioTek Cytation 5 imager (Agilent Technologies, Santa Clara, CA, USA).

### 2.9. Western Blot Analysis

Cell lysates were extracted using cold RIPA buffer (Cell Biolabs Inc., San Diego, CA, USA; Cat. No. AKR-190) with protease inhibitor and quantified using Pierce BCA Protein Assay Kit (Thermo Fisher Scientific, Waltham, MA, USA; Cat. No. PI23225). Denatured lysates were subjected to SDS-PAGE, transferred to PVDF membranes, and blocked with 3% BSA (Sigma-Aldrich, St. Louis, MO, USA; Cat. No. A8022) for 1 h. The membranes were then incubated with CD44 primary antibody (Thermo Fisher Scientific, Waltham, MA, USA; Cat. No. MA5-13890) at a dilution of 1:1000 and incubated with the membrane overnight at 4 °C in 1% BSA in TBST. Beta-tubulin primary antibody (Santa Cruz Biotechnology, Dallas, TX, USA; Cat No. sc9104) was incubated overnight at 4 °C with the diluted 1% BSA in TBST buffer. HRP conjugated secondary anti-mouse antibody (Santa Cruz Biotechnology, Dallas, TX, USA; Cat. No. sc516102) against CD44 and anti-rabbit antibody (Santa Cruz Biotechnology, Dallas, TX, USA; Cat. No. sc2357) against β-tubulin were used at a concentration of 1:5000 and incubated at room temperature for 1 h. Pierce ECL (Thermo Fisher Scientific, Waltham, MA, USA; Cat. No. 32106) was used for chemiluminescence. Images were acquired using an Amersham Imager 600 (GE Healthcare Life Sciences, Chicago, IL, USA).

### 2.10. Statistical Analysis

All the experiments were conducted with three independent biological repetitions. The evaluation of data, calculation of mean values, and standard deviation were performed utilizing Microsoft Excel. Statistical significance (*p* ≤ 0.05) was assessed through an unpaired, two-tailed Student’s *t*-test. Error bars indicate the standard deviation derived from three biological replicates.

## 3. Results and Discussion

### 3.1. TIP60 Drives Cell Proliferation and Negatively Regulates EMT in CRC

**Figure 1:**
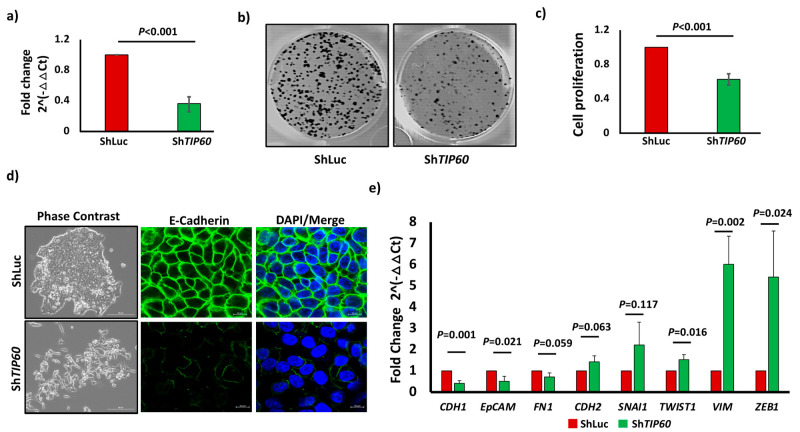
*TIP60* is essential for cellular proliferation and epithelial phenotypes in CRC. (**a**) Bar chart showing qPCR result for *TIP60* expression in HCT116 control (ShLuc) and *TIP60*-depleted (Sh*TIP60*) cells. (**b**) Colony formation assay in ShLuc and Sh*TIP60* cells. (**c**) Bar chart showing cell proliferation after *TIP60* depletion. (**d**) Phase-contrast image of HCT116 ShLuc, Sh*TIP60* cells (**left panel**), Scale bars: 200 μm and immunofluorescence staining of E-cadherin (**right panel**), Scale bars: 10 μm. (**e**) Bar chart showing qPCR results in fold change for EMT genes in HCT116 ShLuc, Sh*TIP60* cells. Error bars indicate standard deviation for three biological repetitions and *p*-value by unpaired two-tailed Student’s *t*-test.

To investigate the functional role of TIP60 in CRC, we utilized sh*TIP60* to deplete *TIP60* expression in CRC cell lines. We achieved a knockdown efficiency of 64% (*p* = 0.0002) for HCT116 cell lines, as analyzed by qPCR (Figure 1a). To identify the role of TIP60 in CRC proliferation, we performed a colony formation assay (CFA) by seeding 1000 cells in 6-well plates and incubating them for 11 days. The CFA shows a significant reduction in cell proliferation (Figure 1b), which was further confirmed by the absorbance assay showing reduced cell proliferation of 37% (*p* = 0.0005) in *TIP60*-depleted (Sh*TIP60*) cells (Figure 1c). This finding is in agreement with previous studies in squamous cell carcinoma [21] and prostate cancer [22], which suggests that *TIP60* is necessary for cellular proliferation in CRC. Since *TIP60* regulates cellular invasion and metastasis, we looked at the cell morphology to find out if there are any changes between control (ShLuc) and *TIP60*-depleted (ShT*IP60*) cells. The phase-contrast image shows that depletion of *TIP60* results in a phenotypic change from compact cell colonies to random spindle networks in cultures, suggesting that *TIP60*-depleted cells undergo EMT. We also tested additional conditions to understand the role of *TIP60* in CRC by overexpressing the *TIP60* wild-type (*TIP60*_WT) and mutating its catalytic domain (*TIP60*_KD), which is essential for its lysine acetyltransferase activity. The phase-contrast image shows a compact colony in HCT116 *TIP60*_WT cells, whereas in *TIP60*_KD, it resembles a mesenchymal phenotype, suggesting a dominant negative effect due to mutation in the catalytic domain (Figure S1a). To further confirm *TIP60*-depleted (ShT*IP60*) cells undergoing EMT, we performed immunofluorescence staining of E-cadherin and qPCR analysis for EMT genes in HCT116 ShLuc and Sh*TIP60* cells. E-cadherin staining in ShLuc showed linear junctional staining, whereas in Sh*TIP60* cells, there was a loss of junctional staining with a punctate pattern, confirming that *TIP60*-depleted cells undergo EMT (Figure 1d, right panel). qPCR analysis for EMT genes revealed that epithelial markers such as E-cadherin (*CDH1*) (*p* = 0.0013) and *EpCAM* (*p* = 0.0218) were significantly downregulated. In contrast, mesenchymal markers such as *TWIST1* (*p* = 0.0163), Vimentin (*VIM*) (*p* = 0.0028), and *ZEB1* (*p* = 0.0243) were significantly upregulated (Figure 1e). In breast cancer, it has been demonstrated that *TIP60* is essential for the epithelial phenotype [15]. The findings of our study indicate that the loss of epithelial phenotype is a consequence of the depletion of *TIP60*, which suggests that it plays a role as an epithelial gatekeeper in CRC. Together, these data suggest that *TIP60* is essential for cell proliferation and epithelial phenotypes in CRC.

### 3.2. TIP60 Modulates Cancer Stem Cell Characteristics in CRC

**Figure 2:**
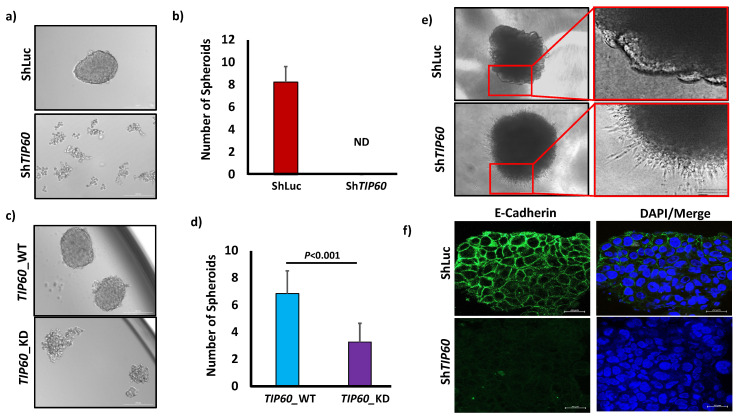
*TIP60* regulates colorectal cancer stemness. (**a**) Phase-contrast image of HCT116 ShLuc and Sh*TIP60* cells in 3D culture, Scale bars: 200 μm. (**b**) Bar chart showing number of spheroids formed per 100 cells. (**c**) Phase-contrast image of HCT116 *TIP60*_WT and *TIP60*_KD cells in 3D culture, Scale bars: 200 μm. (**d**) Bar chart showing number of spheroids formed per 100 cells. (**e**) Phase-contrast image of control (ShLuc) and *TIP60*-depleted (Sh*TIP60*) cells cultured in 3D BME invasion matrix, Scale bars: 1000 μm. (**f**) Immunofluorescence staining of E-Cadherin in 3D culture of HCT116 control (ShLuc) and *TIP60*-depleted (Sh*TIP60*) cells, Scale bars: 20 μm. ND, not detected. Error bars indicate standard deviation for three biological repetitions, with *p*-value by unpaired two-tailed Student’s *t*-test.

To determine *TIP60’s* functional significance in regulating CRCSCs, we performed a spheroid formation assay by seeding 100 cells per well of ShLuc and Sh*TIP60* cells in 96-well ultra-low attachment plates. Spheroid formation assay shows that *TIP60*-depleted cells completely abrogate sphere-forming ability in 3D culture (Figure 2a,b). It has been shown that *TIP60* is involved in stem cell function [16,17]. In an in vitro condition, sphere-forming ability is one of the assays for determining the self-renewal and multipotency of CSC subpopulations inside tumors or cancer cell lines, suggesting the importance of *TIP60* in regulating CRC stemness. To understand the role of *TIP60* in CRC stemness, we also performed a spheroid culture of *TIP60*_WT and *TIP60*_KD. Spheroid formation assay in *TIP60*_WT shows no significant differences compared to ShLuc cells. However, quantification shows that *TIP60*_WT forms bigger spheroids than ShLuc. In contrast, *TIP60*_KD shows a reduced number of spheroid (*p* = 0.0009) formations, smaller in size, indicating the partial loss of *TIP60* functions (Figure 2c,d). This suggests that *TIP60* plays a crucial role in CRC stemness. The 2D culture in Figure 1d,e shows that *TIP60*-depleted cells undergo EMT. To find EMT regulation in the 3D culture condition, we seeded 200 cells per well in 96-well round-bottom ultra-low attachment plates, resuspended in a spheroid-formation extracellular matrix (ECM). Since Sh*TIP60* fails to form spheroids in 3D culture, after seeding 200 cells per well, we spun down the cells at 1500 rpm for 5 min and incubated them for 3 days at 37 °C in sphere-forming conditioned media so the cells could assemble to form aggregates. Our results show that control (ShLuc) cells fail to invade in a 3D invasion matrix, whereas *TIP60*-depleted (Sh*TIP60*) cells infiltrate in 3D culture (Figure 2e), suggesting *TIP60* is involved in maintaining the epithelial phenotype. Moreover, immunofluorescence labeling of E-cadherin in 3D culture (Figure 2f) exhibits a linear junctional staining similar to that shown in 2D culture, confirming the role of *TIP60* in cellular migration and invasion. These data suggest that TIP60 regulates CRC stemness and inhibits cellular invasion in CRC.

### 3.3. The TIP60-CD44 Axis Regulates Cancer Stem Cell Properties

**Figure 3:**
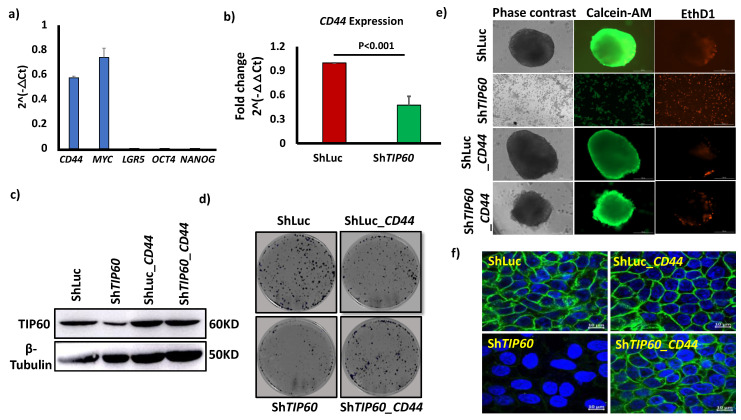
The *TIP60*-*CD44* axis regulates cancer stemness. (**a**) Bar chart showing the expression of *CD44*, *MYC*, *LGR*5, *OCT4*, and *NANOG* normalized to the housekeeping gene (*ACTB* and *GAPDH*) in HCT116 cells grown in 3D. (**b**) Bar chart showing *CD44* expression level in control (ShLuc) and *TIP60*-depleted (ShT*IP60*) cells. (**c**) Western blot data showing the TIP60 expression in ShLuc, Sh*TIP60*, ShLuc_*CD44*, and Sh*TIP60*_*CD44*, β-tubulin used as an internal control. (**d**) Colony formation assay in HCT116 ShLuc, Sh*TIP6*0, ShLuc_*CD44*, and Sh*TIP60*_*CD44* cells. (**e**) Phase-contrast image of HCT116 ShLuc, Sh*TIP60*, ShLuc_*CD44*, and Sh*TIP60*_*CD44* spheroid culture (**left panel**) and CalceinAM and EthD1 staining of HCT116 ShLuc, Sh*TIP60*, ShLuc_*CD44*, and Sh*TIP60*_*CD44* spheroids (**right panel**), Scale bars: 200 μm. (**f**) Immunofluorescence staining of E-cadherin in HCT116 ShLuc, Sh*TIP60*, ShLuc_*CD44*, and Sh*TIP60*_*CD44* cells. UD, undetected, Scale bars: 20 μm. Error bars indicate the standard deviation for three biological repetitions and the *p*-value by unpaired two-tailed Student’s *t*-test.

To have a mechanistic insight on how *TIP60* regulates CRC stemness and to identify downstream targets for *TIP60*, we selected genes that are involved in the regulation of pluripotency, self-renewal, and proliferation in embryonic stem cells/adult stem cells and cancer stem cells such as *LGR5* [23], *MYC* [24], *OCT4* [25], *NANOG* [26], and *CD44* [19] (Figure 3a). qPCR results shows that *CD44* (*p* = 0.0011) (Figure 3b) and *MYC* (*p* = 0.0038) (Figure S1b) were significantly downregulated in *TIP60*-depleted cells (Sh*TIP60*) in 3D culture, whereas the expression of *LGR5*, *OCT4*, and *NANOG* was undetected. Since CD44 is a marker for CSCs across various solid tumors [19], we were interested in how *TIP60* downregulation changes *CD44* expression level and whether *CD44* acts as a downstream target for *TIP60*. The role of *MYC* and *TIP60* has already been established, where *MYC* recruits *TIP60* to chromatin [27]. Meanwhile, the *TIP60*-*CD44* axis has not yet been explored. To identify the role of CD44 in CRC, we overexpressed *CD44* by transfecting *CD44* full-length in HCT116 control (ShLuc) and *TIP60*-depleted (Sh*TIP60*) cells. Western blot results show that overexpression of CD44 upregulates TIP60 expression levels (Figure 3c). To identify the role of the *TIP60*-*CD44* axis in CRC cell proliferation, we performed a colony formation assay that shows *CD44* overexpression restores cell proliferation in HCT116 cells (Figure 3d and S1c). To further confirm the role of *CD44* in cancer stemness, we performed a spheroid formation assay. Overexpression of *CD44* in *TIP60*-depleted (Sh*TIP60*) cells restores spheroid formation efficiency (Figure S1d,e), suggesting that the *TIP60*-*CD44* axis maintains the CSC property. We also performed CalceinAM (stains live cells) and EthD1 (stains dead cells) staining. CalceinAM and EthD1 staining show that *TIP60*-depleted cells (Sh*TIP60*) fail to survive in 3D culture (Figure 3e), suggesting that *TIP60*-depleted cells undergo anoikis in 3D culture. Furthermore, immunofluorescence staining (Figure 3f) and the phase-contrast image (Figure S1f) also show that *TIP60*-depleted cells overexpressing *CD44* (Sh*TIP60*_*CD44*) undergo colony compaction, suggesting an EMT reversal phenotype. Taken together, these data suggest that the *TIP60*-*CD44* axis regulates CRC stemness.

Our findings reveal that TIP60 regulates CRC stemness, cell proliferation, epithelial phenotype, and cellular invasion. Our results are consistent with the knockdown of *TIP60* in non-small-cell lung cancer, showing reduced cell proliferation, migration, and invasion [28]. It has also been shown that in CRC, *TIP60* expression is downregulated (13%) in tumor samples with peritoneal dissemination, distant metastasis, and a higher stage of TNM classification [29]. Similarly, in gastric cancer, where *TIP60* is downregulated in 61% of specimens, it correlates with tumor invasion and lymph node metastasis [30]. These data suggest that TIP60 plays a critical role during tumor initiation and progression, but its downregulation may occur during the transition to a metastatic and advanced stage of CRC. In breast cancer, TIP60 acts as a tumor suppressor gene, and its loss induces genomic instability, leading to the development of cancer [31]. These studies highlight that *TIP60* is a dynamic epigenetic regulator that may induce tumor-promoting and tumor-suppressing actions. In summary, this study reveals that TIP60 regulates cancer stem cells’ properties, cellular proliferation, and EMT in CRC. Targeting *TIP60* will offer a novel therapeutic approach against CRCSCs.

## Figures and Tables

**Table 1:** PCR primer sequences.

Genes	Forward 5′ to 3′	Reverse 5′ to 3′
*TIP60*	AATGTGGCCTGCATCCTAAC	TGTTTTCCCTTCCACTTTGG
*CD44*	CACATATTGCTTCAATGCCTCAG	CCATCACGGTTGACAATAGTTATG
*MYC*	TCAAGAGGTGCCACGTCTCC	TCTTGGCAGCAGGATAGTCCTT
*OCT4*	GGAGTCTGGAGACCATGTTTCTG	GAACCATACTCGAACCACATCCTT
*LGR5*	TTCAATCCCTGCGCCTAGAT	TGCAGGCCGCTGAAACA
*NANOG*	TGGAAGCCACTAGGGAAAGC	TGGAGTCACACACTAGTTCACGAATAA
*CDH1*	TTACTGCCCCCAGAGGATGA	TGCAACGTCGTTACGAGTCA
*EpCAM*	GCTGGCCGTAAACTGCTTTG	ACATTTGGCAGCCAGCTTTG
*FN1*	AACCCTTCCACACCCCAATC	ACTGGGTTGCTGACCAGAAG
*CDH2*	CCGGTTTCATTTGAGGGCAC	TCCCTCAGGAACTGTCCCAT
*SNAI1*	TCTTTCCTCGTCAGGAAGCC	GATCTCCGGAGGTGGGATGG
*SNAI2*	CTCCTCATCTTTGGGGCGAG	CTTCAATGGCATGGGGGTCT
*TWIST1*	TCGGACAAGCTGAGCAAGATT	GCAGCTTGCCATCTTGGAGT
*VIM*	CTGCCAACCGGAACAATGAC	CATTTCACGCATCTGGCGTT
*ZEB1*	AGGATGACCTGCCAACAGAC	CTTCAGGCCCCAGGATTTCTT
*ACTB*	CCAGATCATGTTTGAGACCTTCAAC	CCAGAGGCGTACAGGGATAGC
*GAPDH*	CAGCCTCAAGATCATCAGCA	TGTGGTCATGAGTCCTTCCA

## Data Availability

All the data are available from the corresponding authors upon request.

## Supplementary Materials

The following supporting information can be downloaded at https://www.mdpi.com/article/10.3390/cells14100686/s1, Figure S1. (a) Phase-contrast image of HCT116 *TIP60*_WT and *TIP60*_KD cells. (b) Bar chart showing *MYC* expression in HCT116_ShLuc and HCT116_Sh*TIP60* cells. (c) Bar chart showing cell proliferation in HCT116_ShLuc, HCT116_*ShTIP60*, HCT116_ShLuc_*CD44*, and HCT116_Sh*TIP60*_*CD44* cells. (d) Bar chart showing the number of spheroids formed per 100 cells in HCT116_ShLuc, HCT116_Sh*TIP60*, HCT116_ShLuc_*CD44*, and HCT116_Sh*TIP60*_*CD44* cells. (e) Phase-contrast image of the spheroid in HCT116_ShLuc, HCT116_Sh*TIP60*, HCT116_ShLuc_*CD44*, and Sh*TIP60*_*CD44* cells, Scale bars: 200 μm. (f) Phase-contrast image of HCT116_ShLuc, HCT116_Sh*TIP60*, HCT116_ShLuc_*CD44*, and Sh*TIP60*_*CD44* cells, Scale bars: 200 μm. Error bars indicate the standard deviation for three biological repetitions, and the *p*-value is determined by unpaired two-tailed Student’s *t*-test (NS, not significant; ND, not detected).

## Abbreviations

The following abbreviations are used in this manuscript:

BME	Basement membrane extract
CalceinAM	Calcein Acetoxymethyl Ester
CAMs	Cell adhesion molecules
CRC	Colorectal cancer
CRCSCs	Colorectal cancer stem cells
CSCs	Cancer stem cells
Ct	Threshold cycle
DAPI	4′,6-diamidino-2-phenylindole
DMEM	Dulbecco’s Modified Eagle Medium
ECL	Enhanced chemiluminescence
ECM	Extracellular matrix
EMT	Epithelial–mesenchymal transition
ESCs	Embryonic stem cells
EthD1	Ethidium Homodimer-1
FBS	Fetal Bovine Serum
HRP	Horseradish peroxidase
HSCs	Hematopoietic stem cells
KAT5	Lysine acetyltransferase 5
MYST	Moz, Ybf2/Sas3, Sas2, and TIP60
PVDF	Polyvinylidene difluoride
RT-qPCR	Quantitative real-time PCR
SDS	Sodium dodecyl sulfate
SDS-PAGE	Sodium dodecyl sulfate-polyacrylamide gel electrophoresis
TIP60	HIV-1 Tat-interactive protein
