# Supplementary material for: The TIP60-CD44 Axis Modulates Colorectal Cancer Stemness
Source: Cells. 2025 May 9;14(10):686. doi: 10.3390/cells14100686 (PMC12110716; doi:10.3390/cells14100686)
Supplement: Supplementary file 1 [file cells-14-00686-s001.zip › cells-3529121-supplementary.pdf]

cells

MDPI

Brief Report

The TIP60-CD44 Axis Modulates Colorectal Cancer Stemness

Asad Mohammad, Sudhakar Jha *

College of Veterinary Medicine, Department of Physiological Sciences, Oklahoma State University, Stillwater, OK 74078; moasad@okstate.edu(M.A.)
* Correspondence: sjha@okstate.edu; Tel.: 1-405-744-4409

Academic Editor: Giovanni Amabile

Received: 27 February 2025
Revised: 1 May 2025
Accepted: 5 May 2025
Published: 9 May 2025

Citation: Mohammad, A.; Jha, S. The TIP60-CD44 Axis Modulates Colorectal Cancer Stemness. *Cells* **2025**, *14*, 686. https://doi.org/10.3390/cells14100686

Copyright: © 2025 by the authors. Licensee MDPI, Basel, Switzerland. This article is an open access article distributed under the terms and conditions of the Creative Commons Attribution (CC BY) license (https://creativecommons.org/licenses/by/4.0/).

Supplementary Figure

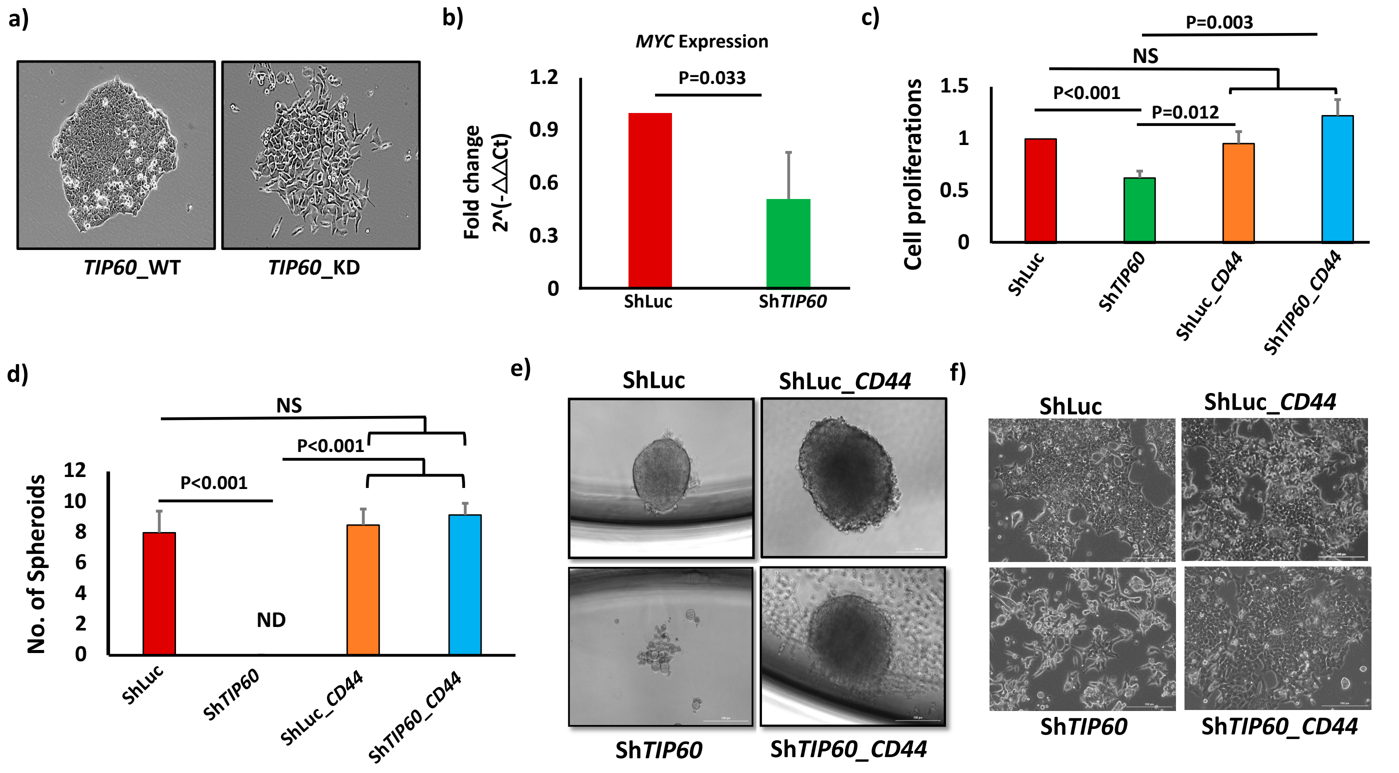

Figure S1. a) Phase contrast image of HCT116 *TIP60*_WT and *TIP60*_KD cells. b) Bar chart showing *MYC* expression in HCT116_ShLuc HCT116_Sh*TIP60* cells. c) Bar chart showing cell proliferation in HCT116_ShLuc HCT116_Sh*TIP60*, HCT116_ShLuc_*CD44*, and HCT116_*ShTIP60_CD44* cells. d) Bar chart showing the number of spheroids formed per 100 cells in HCT116_ShLuc HCT116_Sh*TIP60*, HCT116_ShLuc_*CD44*, and HCT116_Sh*TIP60_CD44* cells. e) Phase contrast image of spheroid in ShLuc, Sh*TIP60*, HCT116_ShLuc_*CD44*, and Sh*TIP60_CD44* cells, Scale bars: 200 µm. f) Phase contrast image of ShLuc, Sh*TIP60*, HCT116_ShLuc_*CD44*, and Sh*TIP60_CD44* cells, Scale bars: 200 µm. Error bars indicate the standard deviation for three biological repetitions, with *p*-value by unpaired two-tailed Student's t-test, (NS- not significant, ND- not detected).
